# From the bench to the market: the long, sinuous and rocky road

**DOI:** 10.1590/1678-77572016ed001

**Published:** 2016

**Authors:** Marília Afonso Rabelo Buzalaf

Dear Readers,

It is very pleasant to go to a nice shop and buy a brand-new, innovative product that was just launched into the market. Most people, however, do not realize the long, sinuous and rocky road between the conception of the idea and the launch of the product.

Having the idea, in fact, is the first, most pleasurable and probably the easiest step in this homeric journey. For the true scientists, from time to time, breaking ideas just pop up. Not all good ideas, indeed, will lead to new cutting-edge products. Unfortunately very few scientists have technological views or are associated with "technologists" to make things happen. Even if this is the case, focus, dedication, ability to deal with frustration and lots and lots of patience will be also necessary.

The subsequent step is to prove the concept that the idea fits somehow and, most importantly, adds valuable changes to the currently accepted scientific model. Money and time are basic requirements to transform the idea into sound scientific results. Some scientists will quit. Those who persist will have to face the Editorial Board of some journals that unfortunately are not open-minded enough to welcome ideas that change (or challenge) concepts. This is not the case of the Journal of Applied Oral Science. In the present issue, Hannas, et al.^6^ propose, for the first time, the use of dentifrices to deliver matrix metalloproteinase (MMP) inhibitors to prevent dentin erosion and abrasion. Curiously, 7 years ago, the Journal of Applied Oral Science was the first to accept and publish a paper proposing the use of MMP inhibitors to prevent dentin erosion^12^.

The process of erosion in dentin is more complex than that occurring in enamel, due to the high content of organic matrix in the first. The erosive challenge in dentin initially removes the minerals from the peritubular/intertubular junction. In sequence, the peritubular dentin is degraded, widening the dentin tubuli. Next, a superficial layer of demineralized organic matrix can be detected, followed by a partially demineralized zone and finally by the sound inner dentin^16^. The demineralized organic matrix limits ionic diffusion across the demineralized surface^13^ ^,^ ^14^ and is resistant to mechanical removal by brushing forces^3^ ^,^ ^5^, which reduces the progression of dentin erosion^4^. But as nothing can be perfect, in the oral cavity there are different proteolytic enzymes, such as MMPs that can degrade the demineralized organic matrix^7^ ^,^ ^18^, thus allowing erosion to progress. In the paper by Kato, et al.^12^ it was shown, using an in situ protocol, that daily rinses with green tea, known for its capacity to inhibit MMPs, significantly reduced dentin erosion, opening a new avenue for the prevention of erosion in dentin. That paper had been rejected by some of the most respected dental journals, some of which, later on, published several studies related to the topic^1^ ^,^ ^2^ ^,^ ^8^ ^-^ ^11^ ^,^ ^15^ ^,^ ^17^ ^,^ ^19^.

Going back to the paper by Hannas, et al.^6^ in this issue, we do not know if in the short or distant future we will be able to use a dentifrice containing MMP inhibitors to prevent dentin erosion. There is still a long way to go. If so, the Journal of Applied Oral Science will have contributed an important step to it!
